# Safe storage of methadone in the home - an audit of the effectiveness of safety information giving

**DOI:** 10.1186/1477-7517-2-9

**Published:** 2005-06-29

**Authors:** Roger N Bloor, Rosanna McAuley, Norman Smalldridge

**Affiliations:** 1 Academic Psychiatry Unit, Keele University Medical School, Academic Suite, Harplands Hospital, Hilton Road, Harpfields, ST4 6TH, UK; 2 Edward Myers Centre, Harplands Hospital, Hilton Road, Harpfields, ST4 6TH, UK

## Abstract

**Background:**

Accidental poisoning by methadone occurs, particularly as a result of children ingesting a parent's methadone. Health care professionals have a responsibility to provide information and guidance to methadone users on safe storage of methadone. The objective of the study was to audit the effectiveness of information giving on the safety of methadone consumption, dose measurement and storage, and the effectiveness of sources of advice available for patients.

**Methods:**

The study was undertaken prior to the introduction of a scheme for the supervised consumption of methadone, in the setting of an NHS Methadone clinic serving a district population of 490,000 in the UK. 185 consecutive patients attending a methadone clinic to collect a methadone prescription were the subject of an anonymous survey. Issues of safety of methadone consumption, storage and safety information provisions were assessed. A telephone survey of the community pharmacists dispensing the methadone covered the availability of measuring devices and provision of advice on safety was undertaken.

**Results:**

Methadone was stored in a variety of locations, a cupboard being most frequent. 95 patients (60.1%) had children either living in or visiting their home. All stored their methadone in a bottle with a child resistant lid; the majority measured doses using either the container supplied by the pharmacist or a plastic measure. 126 patients (78%) confirmed that a pharmacist provided a measuring container on their first visit, 24 (15%) were given a measure on every visit to the pharmacist. Advice on safe storage was recalled by 30% of the patients, and advice on measuring methadone by 28%. Methadone was seen as potentially dangerous by 82% of the patients.

**Conclusion:**

The risks resulting from unsafe storage of methadone may be reduced by daily instalment prescribing and provision of measuring containers on request. Recall of provision of information on safety issues is poor and the adoption of a standard policy on provision information should be seen as a priority. A re-audit of safety of storage of methadone is recommended following the introduction of a standard policy on information provision.

## Background

The number of prescriptions per year for methadone in England increased from 425,400 to 1,318,100 between 1990 and 2001 [1]. Prior to the introduction in 1999 of national guidelines in England [2] with regard to supervised methadone consumption, it was common practice for patients to be prescribed take-home methadone from specialist drug clinics or general practitioners which would be dispensed at community pharmacies. Up to 14 days' supply could be prescribed on one prescription and the patient would be allowed to store this at home. In practice, prior to the development of supervised methadone schemes, where patients consume their methadone at the pharmacy and are observed by the pharmacist, many prescribers controlled the amount dispensed by prescribing daily instalments or for 2 to 3 days at a time to reduce the risk of diversion of supplies and the risk of storage of high volumes. Following the introduction of guidelines for supervised methadone, it became the norm within the UK for patients to have daily methadone prescriptions with supervised consumption at the pharmacy for a minimum of 3 months. Providing that they are compliant with treatment, this supervision can then be discontinued and increasing numbers of days' supply can be dispensed to take home. Even within the current supervised schemes, patients will take home at least one day's supply for unsupervised consumption as pharmacies are not in general open on Sundays.

The introduction of national guidelines has produced some changes in the prescribing methods for methadone in the UK. There is still considerable variation in practice between clinicians both in the dose prescribed and the volume dispensed for take-home use [3]

The risks relating to methadone are not confined to those prescribed methadone or to adults using illicit supplies. The storage of methadone at home poses a potential risk to children living with the person prescribed methadone if the supply is not safely stored.

The value of oral methadone prescribing in the treatment of opiate addicts is confirmed in National Clinical Guidelines [2]. Safety issues relating to storage of methadone at home have been well documented [4]. A report regarding the use of babies' feeding bottles as measuring devices for methadone highlighted the risks to children of access to methadone. The study recommended that all doctors who prescribe methadone should ask their patients how they measure their daily dose of methadone[5]. It is clear from current guidance that health care professionals have a responsibility to provide information and guidance to methadone users on safe storage of methadone. [6]. We undertook this audit to evaluate the effectiveness of the sources of advice that those prescribed methadone may have used with regard to safety of storage and measurement of their methadone dose.

## Methods

### Audit criteria and standard setting

The audit followed a standard audit methodology of selecting appropriate criteria and then selecting standards by which to measure success in achieving the criteria.

#### Criteria

The following criteria were selected following a review of the literature.

1. All patients prescribed methadone should recall being given information on safe storage of methadone

2. All patients who take home methadone should have it dispensed in a child resistant container

3. All patients prescribed methadone for home consumption should have an accurate measuring device available

4. All patients who have methadone at home should store it a child resistant container within a safe locked location.

5. All patients where children may have access to methadone should be aware of the particular risks to children.

6. All patients prescribed methadone should be aware of the risks of accidental overdose.

#### Standards

The standard setting was agreed by the audit team using the following principles.

1. Given the high risks posed by accidental methadone overdose criteria 1 – 6 were allocated a 100% standard

### Development of survey instruments

We devised and piloted a questionnaire for anonymous self completion by patients. The content of the questionnaire was planned to cover the following aspects.

• Volumes of methadone prescribed and stored

• Frequency of pick up of prescriptions

• Frequency of doses

• Location of storage

• Measurement of methadone

• Possible access to the stored methadone by children

• Sources of advice on safe storage and measurement

• Appreciation of the possible risks of methadone.

A checklist for telephone survey of community pharmacists was devised to collect data on the availability of measuring devices and advice on safety aspects of storage and measurement.

### Subjects

#### Patient survey

Over a period of three days, consecutive patients attending to collect a methadone prescription from the specialist clinic were invited to complete the questionnaire. Of the 185 patients attending, 165 (86.4%) completed the questionnaire.

#### Pharmacist survey

All pharmacists recorded in the clinic register as potentially dispensing methadone to patients attending the clinic were contacted by telephone (n = 48). Of these, 36 were actually dispensing methadone during the period of the patient survey and 35 (97.2%) agreed to complete the telephone survey

## Results

### Data analysis

Closed questions were analysed with descriptive statistics, open questions by content analysis, a χ^2 ^test was used to compare outcomes

### Volume of methadone stored at home

The mean daily methadone dose for the 161 patients was 32 mg, (Range 5 mg to 80 mg, SD = 14.01). Instalment prescribing is the norm within the clinic and 97 patients (63%) reported a daily pick up, 52 (34%) a pick up every 2 days, 3 patients (2%) picked up twice a week whilst 1 patient (<1%) reported a weekly pick up.

Volumes stored at home reflected the range of doses and the type of instalment prescribing. The mean volume stored at home was 51 mg (Range 0 mg to 315 mg, SD= 48.3). Of the 11 patients who reported that they did not store any methadone at home, 2 reported that they stored it about their person, 8 consumed all their methadone in the street after it had been dispensed and 1 gave it to a parent for safekeeping.

### Location of storage

Content analysis of the location of storage revealed a variety of locations (Table 1). A cupboard was the most common storage place 49 (30.6%), 27 (16.8%) stored methadone in the fridge, whilst only 4 patients (2.5%) stored methadone in a medicine cabinet. One patient stored methadone in a wastebin

**Table 1:** Location of storage of methadone in the home

No response	12 (7.50%)
Wardrobe	7 (4.38%)
Kitchen	7 (4.38%)
On person	4 (2.50%)
Medicine cabinet	4 (2.50%)
Shelf	4 (2.50%)
Mum's House	2 (1.25%)
Unspecified	2 (1.25%)
Bathroom	2 (1.25%)
Living room	1 (0.63%)
Bag	1 (0.63%)
Wastebin	1 (0.63%)
Drawer	1 (0.63%)

### Safety of storage

Of the 159 patients who completed the item on security of their place of storage, 43 (27%) acknowledged that other people would have access to their storage place.

The presence of children in the house was assessed in two ways, firstly as an item enquiring as to children resident with the patient and secondly an item regarding children who may visit the patient. From these items a consolidated figure of homes where children may have access to methadone was calculated.

158 patients responded to items on children within the home, of whom 95 (60.1%) had children either living in or visiting the home. An assessment by these 95 patients of the risk of children knowing where the methadone was stored resulted in 5 patients (3.2%) accepting that children would know where they kept their methadone, 10 patients (6.45%) reported that they thought children could find their methadone.

Chi square analysis of methadone storage location and assessment of the ability of others to access the storage site showed no significant difference between the group with children living in or visiting their home and those with no children living in or having access to their home.

### Methadone storage container

Of the 160 patients who completed the questionnaire, 100% stored their methadone in the original pharmacists' container supplied with a child resistant cap.

### Measurement of methadone

An open question with regard to containers used to measure out methadone doses revealed a fairly narrow range of containers to be in use (Table 2). 140 patients (67.5%) used either the container supplied by the pharmacist or a plastic measure supplied by the pharmacist to measure out their methadone; a minority of 5 patients (3.13%) guessed the correct amount without any form of measure.

**Table 2:** Measuring Methadone (n = 160)

Measuring device	Number (%)
Plastic Measure	100 (62.50%)
Pharmacist's dispensing container	40 (25.00%)
Bottle Cap	10 (6.25%)
Guessing Amount	5 (3.13%)
Injection Syringe	3 (1.88%)
Spoon	1 (0.63%)
Cup	1 (0.63%)

The need to measure out methadone occurs when more than one days supply is dispensed, such as at weekends or when patients have a less frequent prescription instalment. The need to measure out methadone also occurs when patients split their daily dose. Responses to the survey indicated that of the 153 who responded to this item, 100 (63%) take their methadone as single dose. The remaining patients split their dose, 52 (34%) taking methadone twice a day, and 4 (3%) taking it three or more times a day.

126 patients (78%) confirmed provision of measuring containers on the first visit to a pharmacist. 24 patients (15%) reported that they were given a measuring container on every visit to the pharmacist and 13 (8%) reported that they were able to request a measuring device when they needed one

### Sources of advice on storage and measurement

Only 49 patients (31%) recalled being given advice on safety of methadone; of those who did recall this advice, it had been given by the Methadone Clinic (41.7%), the local drug agency (27%) or the pharmacist (21%).

Advice on ways to measure out methadone was recalled by 45 patients (28%), this advice had been given by the methadone clinic (40%), the pharmacist (36%) or the local drug agency (18%).

### Knowledge of the risks of methadone

In response to the question " Is methadone dangerous?". 131 patients (82%) replied yes, 25 patients (16%) replied no and 3 (2%) did not respond. None of the patients reported having being involved with any accidental use of methadone.

### Pharmacist survey

Of the 35 pharmacists who participated in the survey, 32 (91%) confirmed that they would provide a measuring device on request. Only 5 (14.3%) provided a measuring device on each attendance.

Advice on storage of methadone had been given by 4 pharmacists (11%) and advice on measuring out methadone by 6 (17%).

The pharmacists were each dispensing for a mean of 5 patients (Range 1 to 20, SD 4.4)

### Audit criteria

Performance on criteria 1 to 6 was measured against the defined standards. The results are summarised in table 3.

**Table 3:** Audit criteria performance

**Criteria**	**Standards**	**Results**
All patients who take home methadone should have it dispensed in a child resistant container	100%	31%
All patients who take home methadone should have it dispensed in a child resistant container	100%	100 %
All patients prescribed methadone for home consumption should have an accurate measuring device available	100%	62.5%
All patients who have methadone at home should store it in a child resistant container within a safe locked location.	100%	2.5%
All patients where children may have access to methadone should be aware of the particular risks to children.	100%	93.6%
All patients prescribed methadone should be aware of the risks of accidental overdose	100%	82%

Only 1 of the standards reached 100% in the sample studied, that being the dispensing of methadone in containers with child resistant caps.

## Discussion

The accidental ingestion of methadone is a well recognised risk of methadone prescribing[7,8] The need to store methadone is increased if prescriptions are dispensed in more than daily instalments. A survey of prescribing to opiate addicts in England and Wales in 1996 showed that up to 36% of prescriptions for methadone were dispensed on a weekly basis [9]. The use of inappropriate storage and measuring containers for methadone, such as babies' bottles by over 25% of patients in Dublin, was perhaps influenced by the fact that over 50 % of prescriptions for methadone in Dublin were dispensed on a weekly basis [5].

The routine supply of measuring containers is not necessary when methadone is dispensed on a daily basis or its consumption is supervised, apart from at weekends and when the patient takes the methadone in divided doses. The pharmacists surveyed in this study were able to provide measuring containers on request and the patients appeared to be aware of this facility and had obtained them when needed.

The provision of advice to patients on safe storage and measurement had been received by a minority of patients. Pharmacists confirmed that they had only given advice to a small proportion of the patients.

The level of advice reported appears to be consistent with that reported by Calman et al [4] in 1996, as do the various locations chosen by patients to store methadone. The patients' responses to the present study do however indicate a high level of awareness of the risks of methadone both to children and to non drug using adults.

The risks associated with methadone storage and measurement can be seen to be reduced by daily instalment prescribing, provision of measuring devices on request and the provision of information on the particular risks of methadone to children.

The risks to children of unsafe storage of medicines is not of course confined to methadone. Studies of accidental poisoning of children from prescribed medications have shown consistently that failure to store medication in a child resistant container in a safe location is a major factor in increasing the risk of accidental poisoning [10,11]

The responsibility for giving advice on these matters does not appear to be allocated to any one agency and our study reveals that many patients do not recall being given such advice. Provision of information on safety issues is poor and the adoption of a standard policy on provision of written information should be seen as a priority.

A survey of community pharmacists in Scotland undertaken by Matheson and Bond [12] indicated that pharmacists providing health promotion advice to drug misusers see verbal advice as being "risky", whereas written information is seen as non-confrontational. The introduction of written information on storage and measurement to be given out at the time of dispensing of methadone may be one possible solution to ensuring that patients remain aware of the risks inherent in irresponsible custody of their methadone and what steps to take to reduce this risk.

Studies of the relative effectiveness of written versus verbal information on patient information retention and subsequent action do not, however, show any advantage of written over verbal presentation[13,14]. The overall view is that providing the information in both forms provides a range of options which may match the patients' preferred mode of receiving information.

## Conclusion

The provision of information on the safe storage of methadone is recalled by a minority of patients, and the vast majority of patients do not store their methadone in a locked cupboard or other secure location.

The audit we have reported will be repeated following the provision of written information to patients in addition to verbal information at the point that they commence their methadone treatment. This will be reinforced at the point where patients transfer from supervised consumption at the pharmacy to home consumption as part of a progressive relaxation of restrictions in more stable patients.

## Competing interests

The author(s) declare that they have no competing interests.

## Authors' contributions

RNB conceived of the audit, devised the methodology and drafted the manuscript

RM supervised the data collection and performed the telephone survey

NS participated in the design of the study and performed the data analysis

All authors read and approved the manuscript
